# Supplementary material for: Calcium/calmodulin-dependent protein kinase IV promotes imiquimod-induced psoriatic inflammation via macrophages and keratinocytes in mice
Source: Nat Commun. 2022 Jul 22;13:4255. doi: 10.1038/s41467-022-31935-8 (PMC9307837; doi:10.1038/s41467-022-31935-8)
Supplement: Supplementary file 1 — Supplementary Information [file 41467_2022_31935_MOESM1_ESM.pdf]

Calcium/calmodulin-dependent protein kinase IV promotes imiquimod-induced psoriatic inflammation via macrophages and keratinocytes in mice

Liang Yong[1,2,3,4,5,#], Yafen Yu[1,2,3,4,5,#], Bao Li[6,#], Huiyao Ge[1,2,3,4,5], Qi Zhen[1,2,3,4,5], Yiwen Mao[1,2,3,4,5], Yanxia Yu[1,2,3,4,5], Lu Cao[1,2,3,4,5], Ruixue Zhang[1,2,3,4,5], Zhuo Li[1,2,3,4,5], Yirui Wang[1,2,3,4,5], Wencheng Fan[1,2,3,4,5], Chang Zhang[1,2,3,4,5], Daiyue Wang[1,2,3,4,5], Sihan Luo[1,2,3,4,5], Yuanming Bai[1,2,3,4,5], Shirui Chen[1,2,3,4,5], Weiwei Chen[1,2,3,4,5], Miao Liu[7], Jijia Shen[7], Liangdan Sun[1,2,3,4,5,*]

[1] Department of Dermatology, the First Affiliated Hospital of Anhui Medical University, Hefei, China.

[2] Institute of Dermatology, Anhui Medical University, Hefei, China.

[3] Key Laboratory of Dermatology (Anhui Medical University), Ministry of Education, Hefei, China.

[4] Inflammation and Immune Mediated Diseases Laboratory of Anhui Province, Hefei, China.

[5] Anhui Provincial Institute of Translational Medicine, Hefei, China.

[6] Integrated Laboratory, School of Basic Medical Sciences, Anhui Medical University, Hefei, China.

[7] Anhui Provincial Laboratory of Microbiology and Parasitology; Department of Microbiology and Parasitology, Anhui Medical University, Hefei, China.

[#] These authors contributed equally to this work.

[*] ***Correspondence to***: Liangdan Sun, email: ahmusld@163.com.

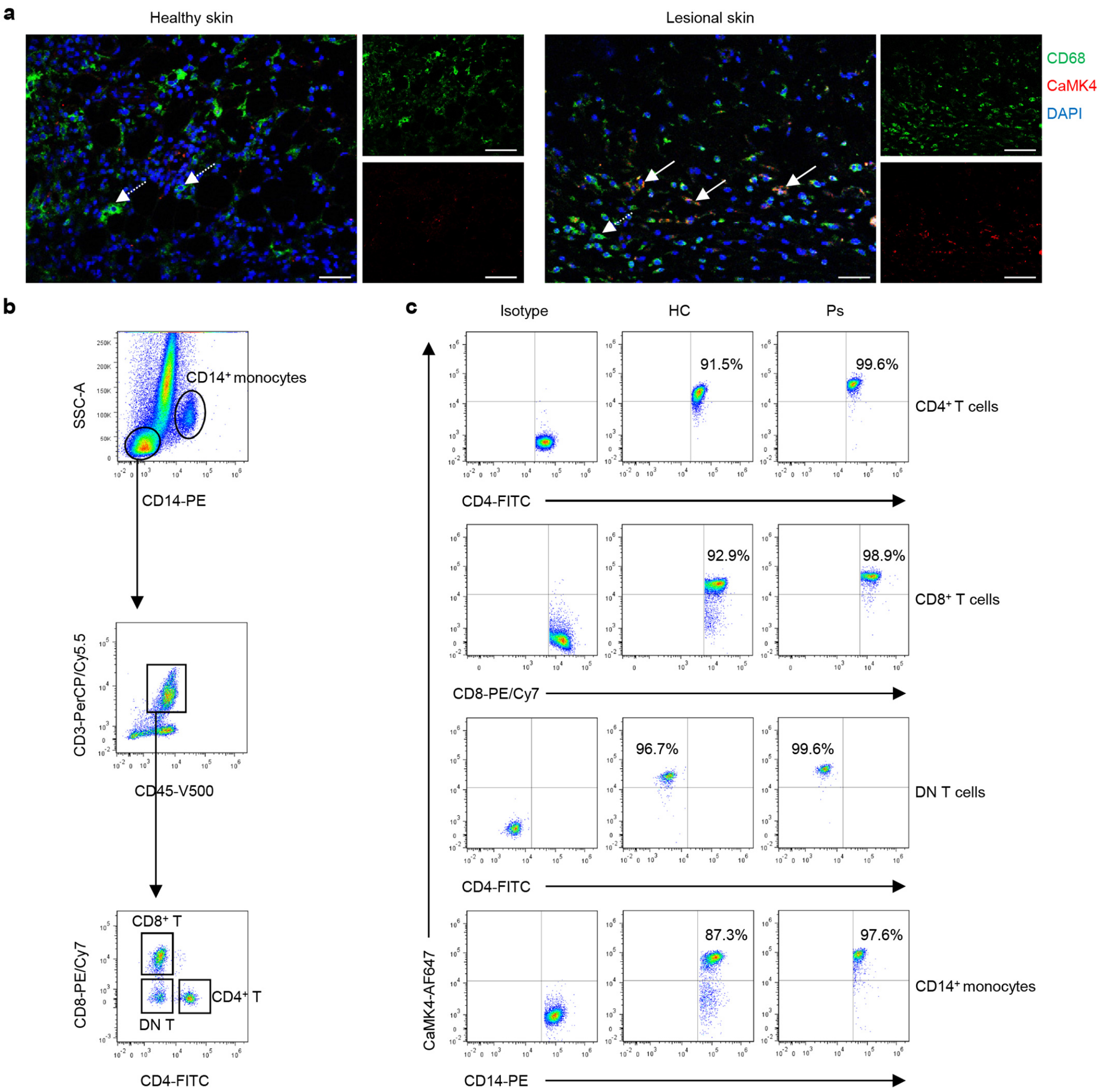

Supplementary Figure 1. CaMK4 expression in human skin tissues, skin macrophages, and peripheral T cell subsets and CD14$^+$ monocytes. a Immunofluorescent microscopy images of human skin sections stained with anti-CD68 (green), anti-CaMK4 (red), and DAPI (blue) (n = 4 per group). The solid arrows show CD68$^+$CaMK4$^+$ cells, and the dashed arrows show CD68$^+$CaMK4$^-$ cells. Scale bar = 50 μm. **b** The gating strategy for the identification of human peripheral T cell subsets and CD14$^+$ monocytes. **c** Representative flow cytometry plots of CaMK4$^+$ cells in peripheral T cell subsets and CD14$^+$ monocytes.

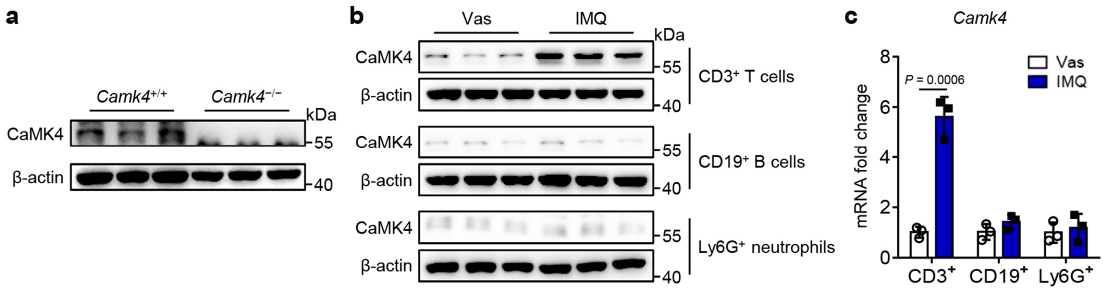

Supplementary Figure 2. Detection of CaMK4 expression. a Western blot analysis of CaMK4 expression in isolated immune cells from the skin of *Camk4*$^{+/+}$ and *Camk4*$^{-/-}$ mice for verifying the specificity of CaMK4 antibody (n = 3 biologically independent samples). **b** Western blot analysis of CaMK4 expression in MACS-sorted CD3^{+} T cells, CD19^{+} B cells, and Ly6G^{+} neutrophils from mouse skin (n = 3 biologically independent samples). **c** Quantitative PCR analysis of *Camk4* expression as indicated (n = 3 biologically independent samples). Five to seven mouse skin tissues were pooled as one sample for MACS sorting, and the purity of sorted cells was > 90% (**b** and **c**). Data are shown as mean ± SD. For (**c**), two-sided unpaired Student's *t*-test. Source data are provided as a Source Data file.

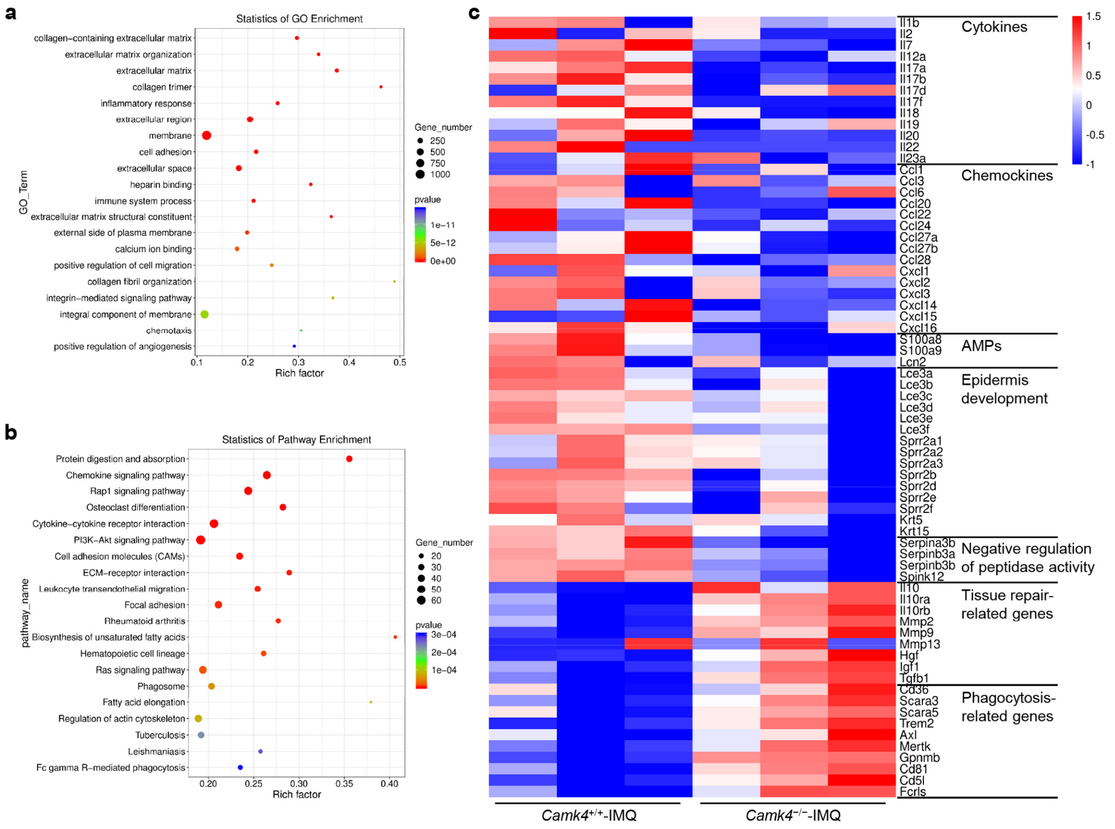

Supplementary Figure 3. Comparison of transcription profile between the whole skin of IMQ-treated *Camk4*$^{+/+}$ and *Camk4*$^{-/-}$ mice. a GO enrichment analysis of differentially expressed genes. **b** KEGG pathway enrichment analysis of differentially expressed genes. **c** Heat map of cytokines, chemockines, AMPs, epidermis development, negative regulation of peptidase activity, tissue repair-related genes, and phagocytosis-related genes in the whole skin of IMQ-treated *Camk4*$^{+/+}$ and *Camk4*$^{-/-}$ mice (n = 3 biologically independent samples).

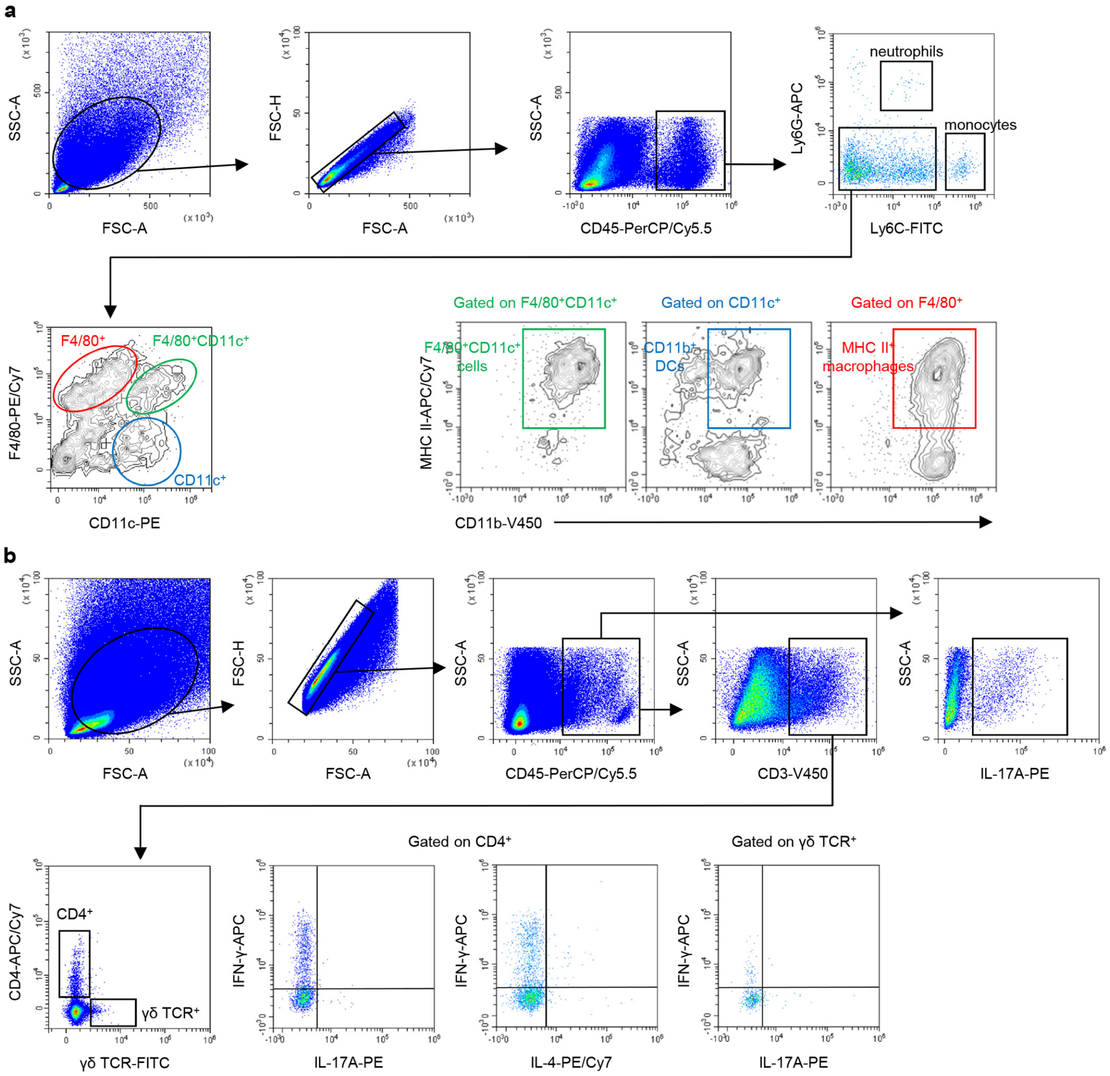

Supplementary Figure 4. The gating strategy for the identification of mouse skin myeloid cell and T cell subsets. a Representative flow cytometry plots show gating strategy to identify mouse skin myeloid cell subsets. **b** Representative flow cytometry plots show gating strategy to identify mouse skin T cell subsets.

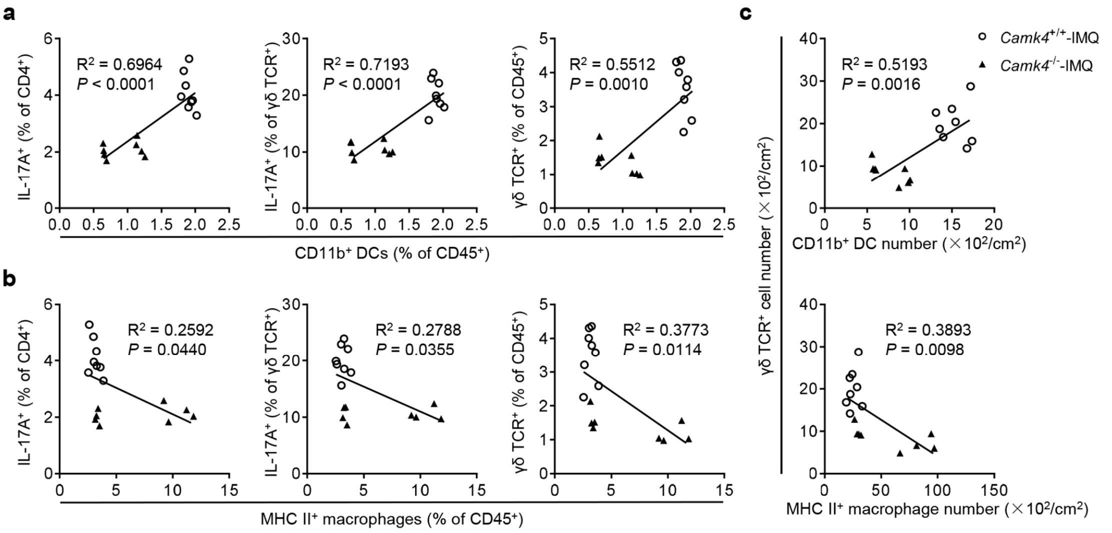

Supplementary Figure 5. Correlation analysis between IL-17A-producing cells and CD11b$^+$ DCs, MHC II$^+$ macrophages in the skin of IMQ-treated *Camk4*$^{+/+}$ and *Camk4*$^{-/-}$ mice. a Linear regression analysis between the percentage of CD11b$^+$ DCs and the percentages of IL-17A$^+$CD4$^+$, IL-17A$^+$γδ TCR$^+$, and γδ TCR$^+$ cells. **b** Linear regression analysis between the percentage of MHC II$^+$ macrophages and the percentages of IL-17A$^+$CD4$^+$, IL-17A$^+$γδ TCR$^+$, and γδ TCR$^+$ cells. **c** Linear regression analysis between the number of γδ TCR$^+$ cells and the numbers of CD11b$^+$ DCs, MHC II$^+$ macrophages. **a**-**d** N = 8 per group from two independent experiments. Data are shown as mean ± SD. For (**a**-**c**), linear regression analysis. Source data are provided as a Source Data file.

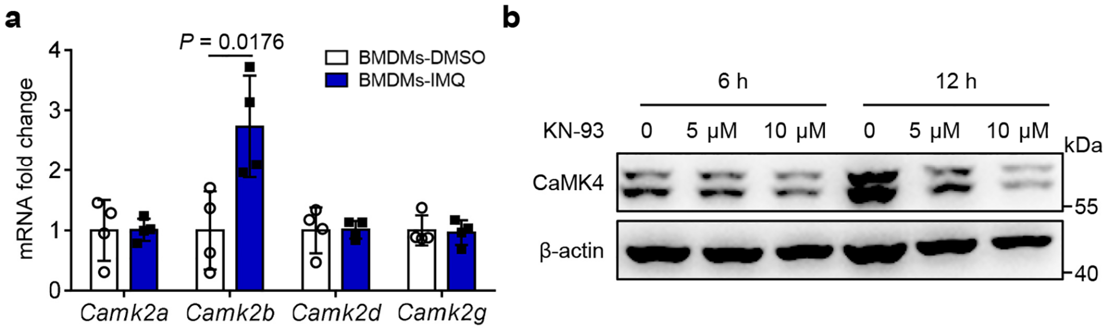

Supplementary Figure 6. The expression of *Camk2* family genes and test of KN-93 inhibition efficiency in BMDMs. a Quantitative PCR analysis of *Camk2* family gene expression in BMDMs (n = 4 biologically independent samples). **b** Western blot analysis of CaMK4 expression as indicated. The experiments in (**b**) were repeated three times with similar results. Data are shown as mean ± SD. For (**a**), two-sided unpaired Student's *t*-test. Source data are provided as a Source Data file.

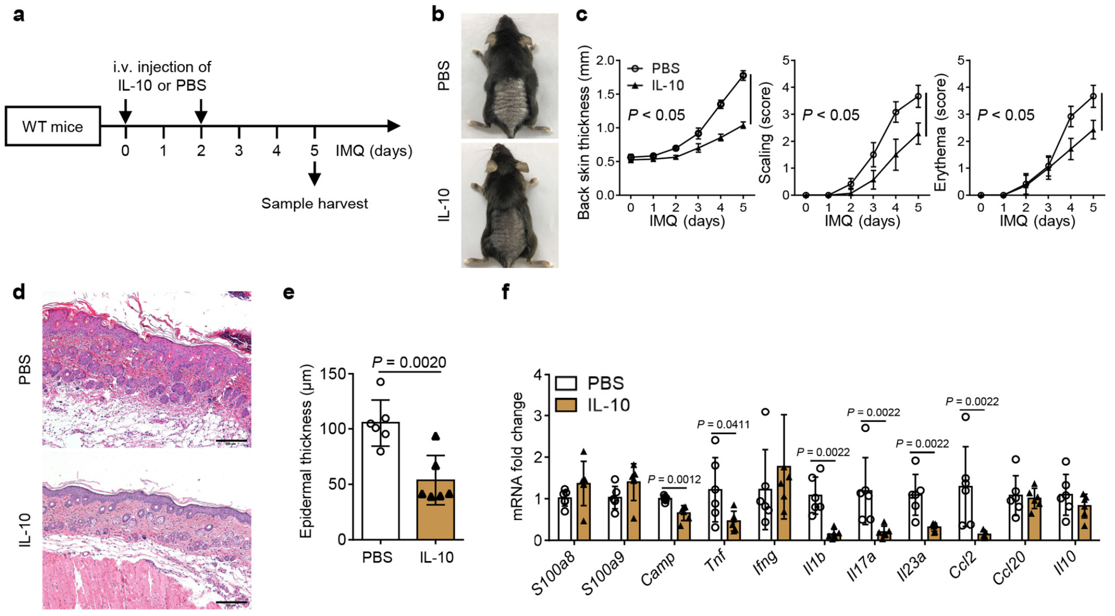

Supplementary Figure 7. Exogenous IL-10 addition alleviates the severity of IMQ-induced psoriatic inflammation. a Schematic representation of the mouse model. WT mice were topically treated with 62.5 mg IMQ on shaved back skin daily for 5 consecutive days. Recombinant mouse IL-10 (1 μg per mouse) or PBS was intravenously injected into each mouse 1 h before IMQ treatment at day 0 and day 2. Samples were harvested at day 5 for subsequent experiments. **b** Representative photos of mouse back skin. **c** Scoring curves of back skin thickness, scaling, and erythema. **d** H&E staining of skin sections. Scale bar = 200 μm. **e** Statistical analysis of epidermal thickness. **f** The expression of pathogenic factors in the skin of IMQ-treated mice as determined by quantitative PCR. **a-f** N = 6 per group from two independent experiments. Data are shown as mean ± SD. For (**c** and **e**), two-sided unpaired Student's *t*-test; for (**f**), two-sided Mann-Whitney test and two-sided unpaired Student's *t*-test. Source data are provided as a Source Data file.

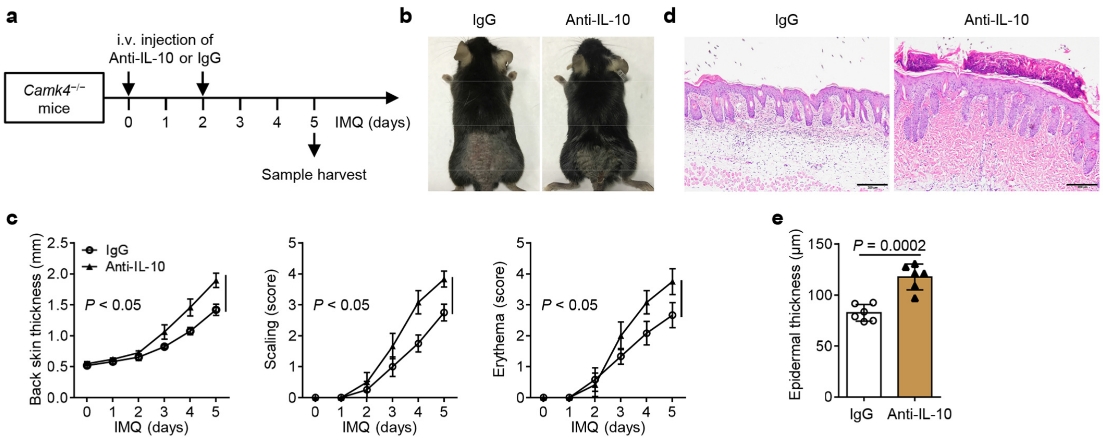

Supplementary Figure 8. Endogenous IL-10 neutralization aggravates the severity of IMQ-induced psoriasis. a Schematic representation of the mouse model. *Camk4*$^{-/-}$ mice were topically treated with 62.5 mg IMQ on shaved back skin daily for 5 consecutive days. Anti-IL-10 (0.2 mg per mouse) or IgG was intravenously injected into each mouse 1 h before IMQ treatment at day 0 and day 2. Samples were harvested at day 5 for subsequent experiments. **b** Representative photos of mouse back skin. **c** Scoring curves of back skin thickness, scaling, and erythema. **d** H&E staining of skin sections. Scale bar = 200 µm. **e** Statistical analysis of epidermal thickness. **a-e** N = 6 per group from two independent experiments. Data are shown as mean ± SD. For (**c** and **e**), two-sided unpaired Student's *t*-test. Source data are provided as a Source Data file.

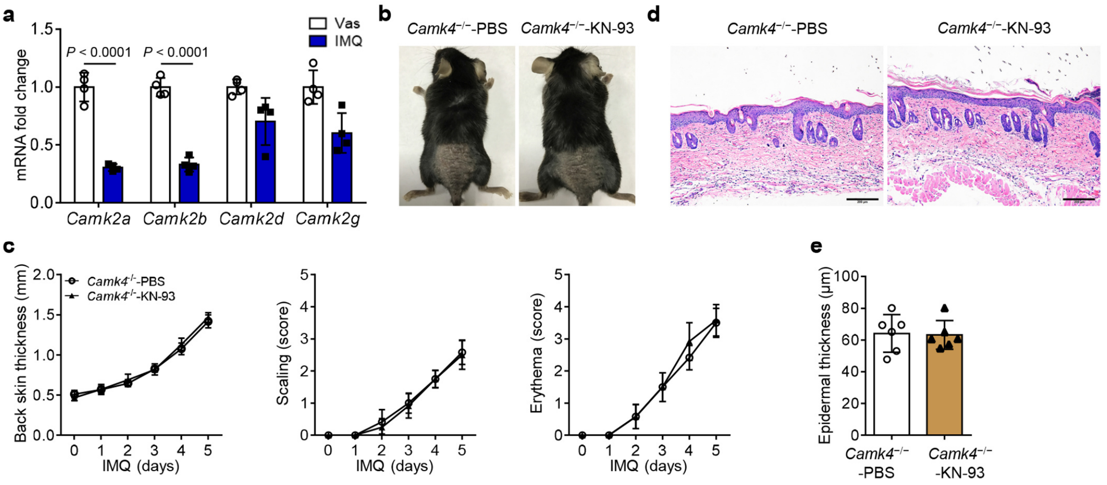

Supplementary Figure 9. CaMK4 inhibitor has no effect on the severity of psoriasis in IMQ-treated *Camk4*$^{-/-}$ mice. a The expression of *Camk2* family genes in the skin of IMQ-treated mice as determined by quantitative PCR (n = 4 biologically independent samples). **b-e** *Camk4*$^{-/-}$ mice were topically treated with 62.5 mg IMQ on shaved back skin daily for 5 consecutive days. KN-93 (0.24 mg per mouse) or PBS was intravenously injected into each mouse 1 h before IMQ treatment at day 0 and day 2. Samples were harvested at day 5 for subsequent experiments. **b** Representative photos of mouse back skin. **c** Scoring curves of back skin thickness, scaling, and erythema. **d** H&E staining of skin sections. Scale bar = 200 μm. **e** Statistical analysis of epidermal thickness. **b-e** N = 6 per group from two independent experiments. Data are shown as mean ± SD. For (**c** and **e**), two-sided unpaired Student's *t*-test. Source data are provided as a Source Data file.

Supplementary Table 1. Partial list of proteins binding to CaMK4 identified using co-IP followed by LC-MS/MS in RAW264.7 cell line

Protein name	Gene name
Tumor rejection antigen peptide pRLib	*Akt1*
cAMP-responsive element modulator	*Crem*
Calmodulin 1	*Calm*
**Adenylate cyclase type 1**	***Adcy1***

Supplementary Table 2. Characteristics of healthy controls and patients with psoriasis

**Expreiment**	**Q-PCR**		**FC**		**CS**		**IHC/IF**	
Group	HC	Ps	HC	Ps	HC	Ps	HC	Ps
Total	24	24	15	12	-	3	7	4
Gender (male/female)	16/8	19/5	8/7	8/4	-	2/1	4/3	2/2
Median age (years)	35	38	40	43	-	38	34	36
PASI	-	8.6 (2.1-31)	-	6.4 (1.6-13.6)	-	6.4 (4.9-8.1)	-	9.7 (3.3-15.6)

Q-PCR, quantitative PCR; FC, flow cytometry; CS, cell sorting; IHC/IF: immunohistochemistry/immunofluorescence

Supplementary Table 3. Primer sequences of mouse

Genes	Forward primer (5'-3')	Reverse primer (5'-3')
Gapdh	GTGTTCCTACCCCCAATGTG	GGTCCTCAGTGTAGCCCAAG
Camk4	GAGAACCTCGTCCCGGATTAC	ACACAATGGATGTAGCACCCC
Camk2a	TGCCTGGTGTTGCTAACCC	CCATTAACTGAACGCTGGAACT
Camk2b	GCACGTCATTGGCGAGGAT	ACGGGTCTCTTCGGACTGG
Camk2d	GATAAACAACAAAGCCAACGTGG	GGATTACAGTAGTTTGGGGCTC
Camk2g	ACCGACGACTACCAGCTTTTC	GCAGCATATTCCTGCGTAGATG
S100a8	AAATCACCATGCCCTCTACAAG	CCCACTTTTATCACCATCGCAA
S100a9	CACCCTGAGCAAGAAGGAAT	TGTCATTTATGAGGGCTTCATTT
Lcn2	ACATTTGTTCCAAGCTCCAGGGC	CATGGCGAACTGGTTGTAGTCCG
Camp	GCTGTGGCGGTCACTATCAC	TGTCTAGGGACTGCTGGTTGA
Tnfa	ACTGGCAGAAGAGGCACTC	CTGGCACCACTAGTTGGTTG
Tgfb1	ACAATTCCTGGCGTTACCTT	AGCCCTGTATTCCGTCTCC
Ifng	ATGAACGCTACACACTGCATC	CCATCCTTTTGCCAGTTCCTC
Il1b	CTGAACTCAACTGTGAAATGC	TGATGTGCTGCTGCGAGA
Il10	GCTCTTACTGACTGGCATGAG	CGCAGCTCTAGGAGCATGTG
Il17a	TTTAACTCCCTTGGCGCAAAA	CTTTCCCTCCGCATTGACAC
Il17f	AACCAGGGCATTTCTGTCCCAC	GGCATTGATGCAGCCTGAGTGT
Il22	ATGAGTTTTTCCCTTATGGGGAC	GCTGGAAGTTGGACACCTCAA
Il23a	ATGCTGGATTGCAGAGCAGTA	ACGGGGCACATTATTTTTAGTCT
Ccl2	CCAGCAAGATGATCCCAATG	TACGGGTCAACTTCACATTC
Ccl20	AATCTGTGTGCGCTGATCCA	CCTTGGGCTGTGTCCAATTC
Cd36	ATGGGCTGTGATCGGAACTG	TTTGCCACGTCATCTGGGTTT
Macro	ACAGAGCCGATTTTGACCAAG	CAGCAGTGCAGTACCTGCC
Trem2	CTGGAACCGTCACCATCACTC	CGAAACTCGATGACTCCTCGG
Axl	ATGGCCGACATTGCCAGTG	CGGTAGTAATCCCCGTTGTAGA
Mertk	CAGGGCCTTTACCAGGGAGA	TGTGTGCTGGATGTGATCTTC
Gpnmb	CATTCCCATCTCGAAGGTGAAA	AAATGGCAGAGTCGTTGAGGA

Cd81	GTGGAGGGCTGCACCAAAT	GACGCAACCACAGAGCTACA
Cd5l	GATCGTGTTTTTCAGAGTCTCCA	TGCAGTCAACCCCTTGAATAAG
Fcrls	ACAGGATCTAAGTGGCTGAATGT	CTGGGTCGTTGCCCTATCTG
Cd80	ACCCCCAACATAACTGAGTCT	TTCCAACCAAGAGAAGCGAGG
Cd86	TGTTTCCGTGGAGACGCAAG	TTGAGCCTTTGTAAATGGGCA
Nos2	GTTCTCAGCCCAACAATACAAGA	GTGGACGGGTCGATGTCAC
Arg1	AACACGGCAGTGGCTTTAACC	GGTTTTCATGTGGCGCATTC
Retnla	CCAATCCAGCTAACTATCCCTCC	CCAGTCAACGAGTAAGCACAG
Chil3	TCTGAAAGACAAGAACACTGAGC	GCAGGTCCAAACTTCCATCC

Supplementary Table 4. Primer sequences of human

Genes	Forward primer (5'-3')	Reverse primer (5'-3')
GAPDH	GTCTCCTCTGACTTCAACAGCG	ACCACCCTGTTGCTGTAGCCAA
CAMK4	GTTCTTCTTCGCCTCTCACATCC	CTGTGACGAGTTCTAGGACCAG
S100A8	ATGCCGTCTACAGGGATGACCT	AGAATGAGGAACTCCTGGAAGTTA
S100A9	GCACCCAGACACCCTGAACCA	TGTGTCCAGGTCCTCCATGATG
CAMP	GACACAGCAGTCACCAGAGGAT	TCACAACTGATGTCAAAGGAGCC
DEFB4A	ATAGGCGATCCTGTTACCTGCC	CATCAGCCACAGCAGCTTCTTG
SPRR2A	GTTCCACAGCTCCACCTTCA	CACAGCCCAGGACTTCCTTT
SPRR2B	ACTGGTTAATCCTGAGACTCCAGC	AGGAGGATATTTCTGCTGGCAC
IL1B	CCACAGACCTTCCAGGAGAATG	GTGCAGTTCAGTGATCGTACAGG
IL6	AAATTCGGTACATCCTCGACGGCA	AGTGCCTCTTTGCTGCTTTCACAC
IL10	GACTTTAAGGGTTACCTGGGTTG	TCACATGCGCCTTGATGTCTG
IL12B	GACATTCTGCGTTCAGGTCCAG	CATTTTTGCGGCAGATGACCGTG
IL23A	GAGCCTTCTCTGCTCCCTGATA	GACTGAGGCTTGGAATCTGCTG
TNFA	CTCTTCTGCCTGCTGCACTTTG	ATGGGCTACAGGCTTGTCACTC
TGFB1	CAATTCCTGGCGATACCTCAG	GCACAACTCCGGTGACATCAA
CCL2	AGAATCACCAGCAGCAAGTGTCC	TCCTGAACCCACTTCTGCTTGG
CCL20	AAGTTGTCTGTGTGCGCAAATCC	CCATTCCAGAAAAGCCACAGTTTT
