# Supplementary material for: Calcium/calmodulin-dependent protein kinase IV promotes imiquimod-induced psoriatic inflammation via macrophages and keratinocytes in mice
Source: Nat Commun. 2022 Jul 22;13:4255. doi: 10.1038/s41467-022-31935-8 (PMC9307837; doi:10.1038/s41467-022-31935-8)
Supplement: Supplementary file 3 — Reporting Summary [file 41467_2022_31935_MOESM3_ESM.pdf]

nature research

Corresponding author(s): Liangdan Sun

Last updated by author(s): Jun 26, 2022

Reporting Summary

Nature Research wishes to improve the reproducibility of the work that we publish. This form provides structure for consistency and transparency in reporting. For further information on Nature Research policies, see our Editorial Policies and the Editorial Policy Checklist.

Statistics

For all statistical analyses, confirm that the following items are present in the figure legend, table legend, main text, or Methods section.

n/a	Confirmed	
☐	☒	The exact sample size (n) for each experimental group/condition, given as a discrete number and unit of measurement
☐	☒	A statement on whether measurements were taken from distinct samples or whether the same sample was measured repeatedly
☐	☒	The statistical test(s) used AND whether they are one- or two-sided<br>*Only common tests should be described solely by name; describe more complex techniques in the Methods section.*
☐	☒	A description of all covariates tested
☐	☒	A description of any assumptions or corrections, such as tests of normality and adjustment for multiple comparisons
☐	☒	A full description of the statistical parameters including central tendency (e.g. means) or other basic estimates (e.g. regression coefficient) AND variation (e.g. standard deviation) or associated estimates of uncertainty (e.g. confidence intervals)
☐	☒	For null hypothesis testing, the test statistic (e.g. F, t, r) with confidence intervals, effect sizes, degrees of freedom and P value noted<br>*Give P values as exact values whenever suitable.*
☒	☐	For Bayesian analysis, information on the choice of priors and Markov chain Monte Carlo settings
☒	☐	For hierarchical and complex designs, identification of the appropriate level for tests and full reporting of outcomes
☒	☐	Estimates of effect sizes (e.g. Cohen's d, Pearson's r), indicating how they were calculated

Our web collection on statistics for biologists contains articles on many of the points above.

Software and code

Policy information about availability of computer code

Data collection	1. Flow cytometry data were collected using a CytoFLEX flow cytometer (Beckman Coulter).<br>2. Quantitative PCR data were collected using a CFX96 Real-Time System (Bio-Rad).<br>3. Western blotting data were collected using a FluorChem FC3 imaging system (ProteinSimple).<br>4. H&E and IHC data were collected using a BX53 upright microscope (Olympus).<br>5. IF data were collected using a TCS SP8 confocal scanning microscope (Leica).
Data analysis	1. Flow cytometry data were processed and analyzed using CytExpert (version 2.4) and FlowJo (version V10).<br>2. Quantitative PCR data were processed and analyzed using Microsoft Excel (version 2013).<br>3. The measurement and quantification of epidermal thickness from H&E were processed and analyzed using cellSens (version 1.5).<br>4. IF data were processed and analyzed using LAS X (version 3.5.1.18803).

For manuscripts utilizing custom algorithms or software that are central to the research but not yet described in published literature, software must be made available to editors and reviewers. We strongly encourage code deposition in a community repository (e.g. GitHub). See the Nature Research guidelines for submitting code & software for further information.

Data

Policy information about availability of data

All manuscripts must include a data availability statement. This statement should provide the following information, where applicable:

- Accession codes, unique identifiers, or web links for publicly available datasets
- A list of figures that have associated raw data
- A description of any restrictions on data availability

The RNA sequencing data generated in this study have been deposited in NCBI Gene Expression Omnibus database under accession code GSE204832 (https://www.ncbi.nlm.nih.gov/geo/query/acc.cgi?acc=GSE204832). Source data are provided with this paper.

Field-specific reporting

Please select the one below that is the best fit for your research. If you are not sure, read the appropriate sections before making your selection.

☒ Life sciences ☐ Behavioural & social sciences ☐ Ecological, evolutionary & environmental sciences

For a reference copy of the document with all sections, see nature.com/documents/nr-reporting-summary-flat.pdf

Life sciences study design

All studies must disclose on these points even when the disclosure is negative.

Sample size	The sample sizes for in vivo and in vitro experiments were determined on the basis of our preliminary/pilot study results. The sample sizes were chosen based on the adequacies to produce statistically differences among the experimental groups using similar experimental conditions on biological replicates in this study.
Data exclusions	No data were excluded from the analyses.
Replication	The detailed number of replicates for each experiment was indicated in figure panels, figure legends or methods, as requested. All attempts at data replication were successful.
Randomization	For the mouse experiments, age-, sex-, and body weight-matched littermates were randomly assigned to different groups in the same experiment. For the cell experiments, cells were randomly assigned to different groups in the same experiment.
Blinding	The investigators were blinded during data collection and analysis where possible, such as PASI of mice, H&E, the measurement and quantification of epidermal thickness, IHC, and IF. The investigators were not blinded for in vitro cell experiments to practically conduct the experiments.

Reporting for specific materials, systems and methods

We require information from authors about some types of materials, experimental systems and methods used in many studies. Here, indicate whether each material, system or method listed is relevant to your study. If you are not sure if a list item applies to your research, read the appropriate section before selecting a response.

Materials & experimental systems

n/a	Involved in the study	
☐	☒	Antibodies
☐	☒	Eukaryotic cell lines
☒	☐	Palaeontology and archaeology
☐	☒	Animals and other organisms
☐	☒	Human research participants
☒	☐	Clinical data
☒	☐	Dual use research of concern

Methods

n/a	Involved in the study	
☒	☐	ChIP-seq
☐	☒	Flow cytometry
☒	☐	MRI-based neuroimaging

Antibodies

Antibodies used	Antibody, Manufacturer, Catalog number, Clone number:<br>V500 anti-human CD45, BD Pharmingen, 560777, HI30;<br>PerCP/Cy5.5 anti-human CD3, BD Pharmingen, 552852, SP34-2;<br>FITC anti-human CD4, BD Pharmingen, 555346, RPA-T4;<br>PE/Cy7 anti-human CD8, BD Pharmingen, 566858, HIT8α;<br>PE anti-human CD14, BD Pharmingen, 555398, M5E2;<br>PerCP/Cy5.5 anti-mouse CD45, BD Pharmingen, 561869, 30-F11;

V450 anti-mouse CD11b, BD Pharmingen, 560456, M1/70;
V450 anti-mouse CD3, BD Pharmingen, 560804, 500A2;
FITC anti-mouse γδ TCR, BD Pharmingen, 561996, GL3;
APC/Cy7 anti-mouse CD4, BD Pharmingen, 561830, GK1.5;
PE anti-mouse CD3, BD Pharmingen, 561824, 145-2C11;
PE anti-mouse CD19, BD Pharmingen, 561736, 1D3;
FITC anti-mouse Ly6C, BioLegend, 128005, HK1.4;
APC anti-mouse Ly6G, BioLegend, 127613, 1A8;
PE anti-mouse CD11c, BioLegend, 117307, N418;
PE/Cy7 anti-mouse F4/80, BioLegend, 123113, BM8;
APC/Cy7 anti-mouse MHC II, BioLegend, 107627, M5/114.15.2;
Alexa Flour 700 anti-mouse Ly6C, BioLegend, 128023, HK1.4;
FITC anti-mouse IL-10, BioLegend, 505005, JES5-16E3;
APC anti-mouse IFN-γ, BioLegend, 505809, XMG1.2;
PE/Cy7 anti-mouse IL-4, BioLegend, 504117, 11B11;
PE anti-mouse IL-17A, BioLegend, 506903, TC11-18H10.1;
PE anti-mouse F4/80, BioLegend, 123109, BM8;
PE anti-mouse Ly6G, BioLegend, 127607, 1A8;
Purified anti-mouse CD16/32, BioLegend, 101302, 93;
Anti-CaMK4, Abcam, ab68218, EP2565AY;
Anti-CD68, Abcam, ab201973, 3F7D3;
Anti-CaMK4, Santa Cruze, sc-55501, H-5;
Anti-ADCY1, Santa Cruze, sc-365350, F-10;
Anti-IL-10, Santa Cruz, sc-52561, JES5-2A5;
Anti-p-Erk1/2, Cell Signaling Technology, 4370T, D13.14.4E;
Anti-Erk1/2, Cell Signaling Technology, 4695T, 137F5;
Anti-p-p38, Cell Signaling Technology, 4511T, D3F9;
Anti-p38, Cell Signaling Technology, 8690T, D13E1;
Anti-p-AKT, Cell Signaling Technology, 4058S, 193H12;
Anti-AKT, Cell Signaling Technology, 4691S, C67E7;
Anti-p-IKKα/β, Cell Signaling Technology, 2697S, 16A6;
Anti-IKKα, Cell Signaling Technology, 61294S, D3W6N;
Anti-p-NF-κB p65, Cell Signaling Technology, 3033T, 93H1;
Anti-NF-κB p65, Cell Signaling Technology, 6956T, L8F6;
Anti-β-actin, Cell Signaling Technology, 4970S, 13E5;
Anti-F4/80, Cell Signaling Technology, 70076S, D2S9R;
InVivoMAb anti-mouse IL-10, Bio X Cell, BE0049, JES5-2A5;
Alexa Fluor 647-conjugated goat anti-rabbit IgG H&L, Abcam, ab150079;
HRP-conjugated goat anti-rabbit IgG (H+L), ZSGB-BIO, ZB-2301;
HRP-conjugated goat anti-mouse IgG (H+L), ZSGB-BIO, ZB-2305;
HRP-conjugated goat anti-rat IgG (H+L), ZSGB-BIO, ZB-2307;
FITC-conjugated goat anti-rabbit IgG (H+L), ZSGB-BIO, ZF-0311;
Rhodamine-conjugated goat anti-rat IgG (H+L), ZSGB-BIO, ZF-0318;
FITC-conjugated goat anti-mouse IgG (H+L), ZSGB-BIO, ZF-0312;
Rhodamine-conjugated goat anti-rabbit IgG (H+L), ZSGB-BIO, ZF-0316.

Validation

All antibodies used in this study were validated for the species and application by the manufacturers on their websites. Quality assessments for all antibodies are provided online by the manufacturers.

Eukaryotic cell lines

Policy information about cell lines

Cell line source(s)

HaCaT and RAW264.7 cells were purchased from the vendor (Chinese Academy of Sciences).

Authentication

All cell lines used in this study were authenticated by the vendor. The authentication certification were provided by the vendor.

Mycoplasma contamination

All cell lines used in this study were tested negative for mycoplasma contamination.

Commonly misidentified lines
(See ICLAC register)

None of the commonly misidentified cell lines were used in this study.

Animals and other organisms

Policy information about studies involving animals; ARRIVE guidelines recommended for reporting animal research

Laboratory animals

Camk4+/+ (or WT), Camk4−/−, Camk4flox/flox, and Lyz2-Cre mice on C57BL/6 background were purchased from GemPharmatech Corporation (Nanjing, China). Camk4flox/flox mice were crossed to Lyz2-Cre mice to create Camk4fl/fl Lyz2-Cre mice. All mice were housed under dark/light cycle of 12 h, ambient temperature of 22-25°C, humidity of 30-70%, and specific-pathogen-free conditions at the Laboratory Animal Center of Anhui Medical University. All mice experiments were performed with 8- to 10-week-old female mice.

Wild animals

The study did not involve wild animals.

Field-collected samples	The study did not involve field-collected samples.
Ethics oversight	All animal experiments were approved by the Institutional Animal Care and Use Committee of Anhui Medical University (Approval number: LLSC20190208) and conformed to the guidelines outlined in the Guide for the Care and Use of Laboratory Animals. All efforts were made to minimize suffering.

Note that full information on the approval of the study protocol must also be provided in the manuscript.

Human research participants

Policy information about studies involving human research participants

Population characteristics	The covariate-relevant population characteristics of the human research participants in every experiment were listed as follow:<br>Quantitative PCR: HC, n = 24, gender (male/female) = 16/8, median age (years) = 35;<br>Ps, n = 24, gender (male/female) = 19/5, median age (years) = 38, PASI = 8.6 (2.1-31).<br>Flow cytometry: HC, n = 15, gender (male/female) = 8/7, median age (years) = 40;<br>Ps, n = 12, gender (male/female) = 8/4, median age (years) = 43, PASI = 6.4 (1.6-13.6).<br>Cell sorting: Ps, n = 3, gender (male/female) = 2/1, median age (years) = 38, PASI = 6.4 (4.9-8.1).<br>IHC/IF: HC, n = 7, gender (male/female) = 4/3, median age (years) = 34;<br>Ps, n = 4, gender (male/female) = 2/2, median age (years) = 36, PASI = 9.7 (3.3-15.6).
Recruitment	Patients with psoriasis were recruited at the clinic and diagnosed with psoriasis vulgaris by two senior dermatologists. Healthy controls were confirmed by physical examination. The written informed consent was obtained from all participants.
Ethics oversight	Human participant study were approved by the Institutional Ethics Committee of Anhui Medical University (Approval number: 20190195) and were performed in accordance with the principles of the Declaration of Helsinki.

Note that full information on the approval of the study protocol must also be provided in the manuscript.

Flow Cytometry

Plots

Confirm that:

☒ The axis labels state the marker and fluorochrome used (e.g. CD4-FITC).

☒ The axis scales are clearly visible. Include numbers along axes only for bottom left plot of group (a 'group' is an analysis of identical markers).

☒ All plots are contour plots with outliers or pseudocolor plots.

☒ A numerical value for number of cells or percentage (with statistics) is provided.

Methodology

Sample preparation	For isolation of immune cells, mouse back skin was separated at the indicated time point, then cut into small pieces and incubated in RPMI 1640 medium containing 1 mg/ml collagenase IV (Sigma), 50 μg/ml DNase I (Sangon Biotech), 10 mM HEPES (Sangon Biotech), and 10% FBS (Gbico) at 37°C for 90 min. Digested skin pieces were passed through a 74-μm nylon mesh and the suspensions were added additional RPMI 1640 to inactivate enzyme activity. Skin leukocytes were isolated by 30% and 70% percoll (GE Healthcare) at 1260g for 20 min. The pellets were resuspended in PBS and the cell number was counted.<br>For human peripheral blood sample staining, anticoagulant blood samples were lysed by ACK Lysis Buffer (0.829% NH4Cl, 0.1% KHCO3, 0.00372% EDTA-2Na, pH 7.2-7.4). After neutralizing and centrifugation, human peripheral leukocytes were acquired.
Instrument	All data were acquired using a CytoFLEX (Beckman Coulter) flow cytometer.
Software	All data were anylazed using CytExpert (version 2.4) software and FlowJo (version V10) software.
Cell population abundance	At least 10000 events were acquired for cells in the defined gate.
Gating strategy	Cells were identified by the FSC-A/SSC-A scatter plot. Doublets were excluded by the FSC-A/FSC-H scatter. Then immune cells were gated on CD45+ cells and different subsets were further gated on a series of markers.

☒ Tick this box to confirm that a figure exemplifying the gating strategy is provided in the Supplementary Information.
